# Electrochemical Direct Determination of Catecholamines for the Early Detection of Neurodegenerative Diseases

**DOI:** 10.3390/s90402437

**Published:** 2009-04-07

**Authors:** Antonella Curulli

**Affiliations:** Institute of Nanostructured Materials (ISMN) CNR Division 2, via del Castro Laurenziano 7, 00161 Rome, Italy; E-Mail: antonella.curulli@ismn.cnr.it; Tel. +390649766743; Fax: +390649766749

**Keywords:** TiO_2_ nanostructured films, chemical sensors, monoamine-neurotransmitters, electrochemical transducers

## Abstract

Smart (Nano) materials with biosensing functions posses enormous potential in development of new generation of stable biosensors, chemical sensors, and actuators. Recently, there is a considerable interest in using TiO_2_ nanostructured materials as a film-forming material since they have high surface area, optical transparency, high bio-compatibility, and relatively good conductivity. In this work, TiO_2_ nanostructured films were used as nanoporous electrodes to study the electron transfer mechanisms of dopamine. epinephrine and norepinephrine, in order to develop a new generation of chemical sensors. The interesting results obtained are described herein and the analytical characterization of these neurotransmitter sensors is reported.

## 1. Introduction

Nanomaterials have attracted tremendeous attention in the research community, due to their unique size-dependent properties [1], which originate from the large contribution of surface atoms to the properties of nanoscale objects and from the size quantization effect [2]. Although the majority of current work on nanomaterials is focused on their optical, electrical, and magnetic properties, and the corresponding devices, a new field of biomedical applications of semiconductor and metal-nanostrcutured oxides has begun to emerge. For instance, II–VI semiconductor and gold nanoparticles modified with antibodies or oligonucleotides can be used as highly stable luminescent and colorimetric tags for immunoanalysis [3]. In this work, we were interested in expanding the scope of possible biomedical applications of nanostructured materials and, in particular, TiO_2_ nanomaterials, which we have identified as potentially useful for neurochemical monitoring. This has become possible due to the utilization of a new type of nanostructured-titania, with particular films having voids and channels of different origin, with pores in the walls of the shells being one of the structural elements. These TiO_2_ nanostructured materials show interesting ion-sieving properties which are fundamental to create electroactive probes using the electrical-charge selectivity and permeability of these-modified electrodes (which depends on the “*surface chemistry properties of these nanomaterials”*) [1] towards charged systems. Considering these properties, in this work we have detected several important biological probes, as dopamine, epinephrine, norepinephrine, i.e., which play a key role during excessive oxidative stress events in humans and in early diagnosis of neurodegenerative diseases.

Focusing on this last point, normal levels of dopamine in the brain allow the usual freedom of movement, whereas excess DA in the brain often creates pleasurable, rewarding feelings and sometime euphoria. One of the most well known and important effects of DA deficiency is Parkinson’s disease (PD) [4,5]. This disease is characterized by degeneration and loss of midbrain *substantia nigra* neurons that produce the neurotransmitter DA, resulting in tremors at rest, inability to initiate or complete movements, muscle rigidity, postural instability and lack of facial expression [4], Neurological investigations have suggested that DA system dysfunction plays a critical role in the diagnosis of PD [4,5] and at the same time, the resulting primary challenge is strictly connected to the measurement of DA and its metabolites under physiological conditions in order to obtain information for a possible early detection of Parkinson’s disease

Moreover, according to other recent clinical studies [6], it seems that the content of ascorbic acid (AA) and dopamine (DA) can be used to assess the amount of oxidation stress in human metabolism, linked to cancer [7], diabetes mellitus [8], and hepatic diseases [9].

However, it is almost impossible to detect this component electrochemically by direct oxidation on a conventional electrode (i.e., glassy carbon, graphite, Au, Pt) because of its high overpotential and because of electrode fouling, poor reproducibility, low selectivity and poor sensitivity. Moreover, the oxidation waves of AA and DA, which coexists with AA in biological liquids, are nearly at the same potential and therefore overlapped, which results in the poor selectivity and reproducibility [10]. Thus, the ability to determine AA or DA selectively in the presence of one another has been a major goal for electroanalytical research and the development of chemical sensors for *in vivo* monitoring. During these last years, many efforts [11] have gone into solving this particular problem but, no satisfactory results have been obtained, especially for clinical, and bio-medical monitoring in real samples. Following this study of developing electrochemical sensors for determination of DA and AA in biological samples, this paper reports the fabrication of TiO_2_ nanostructured –modified Si electrodes based on film deposition on Si plates.

The strong electrocatalytic activity of the TiO_2_ nanomaterials toward DA, and other neurotransmitters, combined with the high ion-selectivity induced from the “*chemistry surface”* properties of TiO_2_ nanostructured oxides, gives a great analytical resolution of anodic peaks of DA and AA by using Differential Pulse Voltammetry (DPV method), which actually represents a widely used technique to detect biological molecules for clinical investigations [12].

Finally, in this work the selective determination of DA in the presence of AA as interferent has been investigated in detail and all the analytical parameters for a correct electroanalytical-characterization of the sensors, were reported and described. In addition, the TiO_2_-modified Si electrodes were applied successfully to the simultaneous determination of DA and AA in their mixtures, in order to develop chemical sensors for *in vivo* monitoring concerning human diseases.

## 2. Experimental

### 2.1. Materials

The present work was carried out in aqueous solutions. Purified water obtained with a Milli-Q (Millipore) water purification system was used as solvent. Dopamine, epinephrine, norepinephrine and ascorbic acid, were obtained from Sigma (St. Louis, MO, USA). All the chemicals from commercial sources were of analytical grade. The solutions were prepared using 0.1 M phosphate buffer (pH 7.4). Before each electrochemical experiment, purified nitrogen gas (N_2_, Rivoira Italy) was used to deoxygenate the solutions. Wafers of Si, p-Si (100) of different shape and size (i.e., minimum diameter of 1 × 1 cm^2^; max. diameter of 5 × 5 cm^2^) were purchased from Merck.

### 2.2. Fabrication of Si-modified Electrodes by Nanostructured TiO_2_ Films

TiO_2_ nanostructured films were deposited by Metal Organic Chemical Vapour Deposition (MOCVD) on Silicon substrates), according to the literature method [13]. After the deposition of nanostructured films, the TiO_2_-modified Si plates (1 cm × 1 cm) were used as working electrode, an Ag/AgCl was used as reference electrode, and a Pt wire as a counter electrode, in a three-electrode electrochemical cell [15]. In order to assure that the working electrodes have the same geometrical area (0.008 ± 0.003 cm^2^), the Si plates coated by the TiO_2_ deposit, were fixed by a Teflon ring with a disk diameter of 1 mm. This Teflon ring equipped with the TiO_2_-modified Si electrode, was terminated with an electrical contact consisting of a copper wire. This was directly connected to an AUTOLAB PGstat/12 potentiostat/galvanostat (Eco Chemie BV, Utrecht, Netherlands) with a metallic crocodile (banana) clip. In addition, a conventional bare Glassy Carbon (GC) electrode (Model Amel CG/492/2, 2 mm diameter, Milan, Italy) was used as working electrode for comparison. All the electrochemical measurements were carried out by AUTOLAB PGstat/12 potentiostat/galvanostat (Eco Chemie BV, Utrecht, Netherlands). All experiments were carried out at room temperature.

## 3. Results and Discussion

### 3.1. Selective Determination of DA in the Presence of AA

A complete material morphological and electrochemical characterisation, using several probes, and their corresponding apparent kinetic constants, K_app_, were described in detail in our previous publications [13–15].

**Figure 1.**
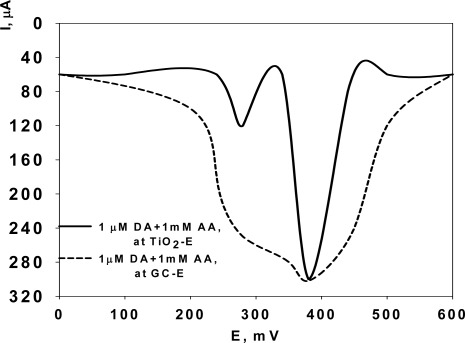
Differential pulse voltammogram of 1 μM DA in 1 mM AA solution, in 0.1 M phosphate buffer, pH 7.4; at GCE (dashed line) and at TiO_2_-modified Si electrode (continous line).

Because the main objective of this investigation was to selectively determine the content of DA in the presence of AA, the electrochemical response of DA and AA binary mixtures at TiO_2_-modified Si electrodes has been investigated by the DPV method with an applied potential E(V) ranging from + 100 mV to + 400 mV *vs*. Ag/AgCl reference electrode. The Differential Pulsed Voltammograms of a binary mixture of 1 μM DA and 1 mM AA in 0.1 phosphate buffer solution at pH 7.4 indicate that the interactions between nanostructured TiO_2_ surfaces and AA and DA lead to the resolution of the overlapped voltammetric wave, observed in the case of conventional GC electrode (Figure 1, dashed line), into well defined peaks at + 280 mV and + 380 mV, corresponding to the oxidation of AA and DA, respectively (Figure 1, continuous line).

The shift of the anodic peak potentials (E_pa_) and the formation of the described well resolved peaks is due to the different interaction of DA and AA with TiO_2_ electrodes, even in the presence of a large amount of AA interferent (i.e., AA concentration ≈ one order of magnitude higher than that of DA in the binary mixture, as in the real biological samples) [12]. The separation between the two peak potentials is 100 mV which is large enough for the selective determination of DA in the presence of AA, and also for the simultaneous determination of DA and AA in their mixtures. An important issue that needs to be taken into consideration is the electrocatalytic oxidation of AA by DA [16–19]. The electrooxidation of DA in the presence of AA results in a homogeneous catalytic oxidation of AA. The oxidized DA, dopamine-*o*-quinone, is chemically reduced by AA to DA, which can be reoxidized at the electrode surface, thus, the oxidation of AA is affected by DA (and “*viceversa”*). Therefore, eliminating this catalytic reaction should be necessary for the accurate determination of DA. For this purpose, considering the predominantly electrostatic nature of the size selectivity in porous TiO_2_ nanostructured films [13–15], one can take advantage of the difference in the ionization state and in the characteristic charge of the permeating ions (e.g., dopamine and ascorbic acid). Provided a sufficient space charge exists inside the nanostructured channels, a Si electrode coated with a TiO_2_ nanostructured layer may behave as if it could be considered simultaneously in the “open” state for dopamine and in the “closed” state for ascorbic acid. This makes possible the selective enhancement of the permeation of positively charged dopamine and retardation of the transport of negatively charged ascorbic acid. All measurements were made at pH 7.4 which represents the physiological value of pH parameter in biological systems. At this pH value, TiO_2_ functionalized nanostructured films are negatively charged and, therefore, the diffusion part of the double layer is made up primarily by cations, as required for the separation of positively charged dopamine and negatively charged ascorbic acid [13–15]. The ratio between the dopamine and ascorbic acid signals changes from 1:3 for a native GC bare surface electrodes [12] to 10:1 for a TiO_2_ nanostructured-modified Si electrode, which give an overall 20-fold enhancement of the selectivity between these substances.

### 3.2. Detection of Neurotransmitters at TiO_2_- modified Si Electrodes: Electroanalytical Performances and Characterization

**Figure 2.**
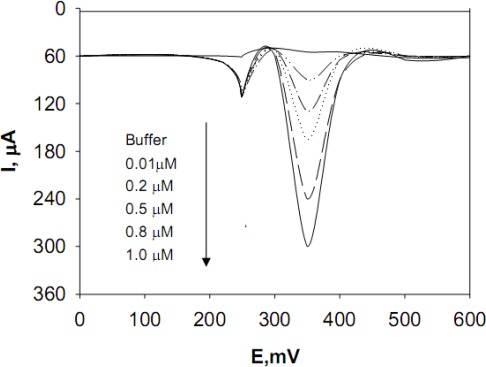
Differential pulse voltammograms of various concentrations of DA (ranging from 0.01 μM to 1 μmol/L) in 1 mM AA solution.

A more detailed evaluation was performed with respect to the analytical performance of the TiO_2_-modified Si electrodes for measuring dopamine, epinephrine, and norepinephine neurotransmitters, which have particular relevance for clinical analysis and biosensor development. The calibrations of these three neurotransmitters at TiO_2_-modified Si electrodes were obtained by Differential Pulse Voltammetry (DPV) with a pulse amplitude of 50 mV; a pulse width of 60 ms; a scan rate of 50 mV/s; a pulse interval of 200 ms; and a sampling time of 20 ms; E_i_ = + 100 mV; and E_f_ = + 400 mV, with an applied potential E(V) ranging from + 300 mV to + 600 mV (see Figure 2, showing the dopamine detection)

Analytical parameters, such as the linearity range, detection limit, reproducibility, and linear regression equations found for the TiO_2_-modified Si electrodes, are reported in Table 1. Overall the results demonstrated the superior behavior of the TiO_2_ nanostructured-modified Si electrodes relative to that of bare GCEs used to measure the same neurotransmitters. In particular, the Si electrodes assembled with TiO_2_ nanostructured films, were able to detect dopamine, epinephrine, and norepinephrine with detection limits an order of magnitude lower, relative to that found for bare GCEs tested under the same conditions.

The detection limit (LOD) was defined as a signal-to-noise ratio of 3 (S/N) = 3. The sensitivity was determined as the slope of calibration curve (μA μM^−1^cm^−2^). The response time of these chemical sensors was evaluated as the time necessary to reach the peak current value, from the current background (I, μA) referred level.

Experimental conditions of DPV measurements were described in Experimental section in the text. For comparison with the TiO_2_-modified Si electrode data given in Table 1, the parameters obtained for dopamine using the reference system, were as given in Table 2.

A bare Si electrode was not used as reference systems because no electrochemistry was observed for DA, epinephrine, and norepinephrine investigated here under the same experimental conditions of TiO_2_-modified Si electrodes, by cyclic voltammetry and DPV methods, consequently a bare GC electrode has been used as reference system.

At a bare GCE, the corresponding values were a detection limit of 3.0 × 10^−7^ M, a linearity range of 3.7 × 10^−7^ – 1.0 × 10^−4^ M, and a linear regression equation Ipa/μA = 1.522 + 0.154 C/μM (correlation coefficient r = 0.99). From these data, the detection limit ranging from to 1.0 × 10^−8^ M, reported here for dopamine at these TiO_2_-modified Si electrodes is very interesting (one order of magnitude compared to GC bare electrodes, the reference system) compared other electrochemical probes described in literature [11,12,16–19]. The electroanalytical performances observed also for epinephrine, and norepinephrine neurotransmitters resulted better than GC bare electrodes, as reported in Table 2, with a detection limit one order of magnitude lower than the corresponding values calculated for a GC bare reference system.

### 3.3. Reproducibility, Operational, and Long Term Stability of the TiO_2_ Based Neurotransmitter Chemical Sensors

The electrodes show high stability toward neurotransmitters in the presence of AA, in terms of operational, and storage (Long-Term) stability. The operational stability of the chemical sensors towards all three neurotransmitters was evaluated keeping these sensors continuously working in a solution of dopamine, epinephrine, and norepinephrine (0.01 μM) solutions, containing 0.01 μM AA, in 0.1 M phosphate buffer solution at pH 7.4, and renewing these solutions every morning. Also in this case the signal, continuously recorded by DPV, showed that during the first 6 hours the analytical signal displayed a 1% decrease, and in the next 24 hours only 2%. The recorded oxidation current, at 72 h (corresponding to 3 days) showed only a 3% decrease vs. the original oxidation current values and over 3 days, 17%.

The storage stability of the chemical sensors in solution was evaluated at + 4 °C and a RT. The response to dopamine, epinephrine, and norepinephrine 0.01 μM solutions containing 0.01 μM AA of 3 sensors, stored at + 4 °C when not in use, and tested every 2 weeks for 10 weeks (over 2 months, 70 days) showed an average decrease of 40% of the initial current value. Another three electrodes were stored at room temperature during 4 weeks and tested every 2 days. The response to dopamine, epinephrine, and norepinephrine 0.01 μM solutions containing 0.01 μM AA of the three sensors was found to be 55 ± 10% of the original one at the end of the period.

**Figure 3.**
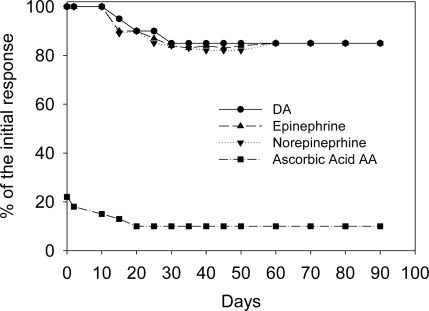
Storage stability in a solution of dopamine, epinephrine, and norepinephrine (0.01 μM) solutions, containing 0.01μM AA, in 0.1 M phosphate buffer solution at pH 7.4.

Figure 3 shows the occasional response obtained for dopamine, epinephrine and norepinephrine (0.01 μM solutions containing 0.01 μmol L^−1^ AA in 0.1 M phosphate buffer, pH 7.0) of 3 different chemical sensors which, during a period over 2 months, have been routinely tested for dopamine, epinephrine and norepinephrine. After a large number of measurements (300) for each sensor, an average response decrease of 40% was observed (see Figure 3). Again several examples of neutransmitters sensors present in literature could be considered [11,12,16–19], but, to our knowledge, the sensor described in this paper was more stable (over three months vs. 15 days maximum).

## 4. Conclusions

As a result of their closely packed morphology and nanoscale dimensions of the pores inside the nanostructures, TiO_2_ films display strong selectivity and they can be used to modify a Si platform for electrode assembly. This quality can be further optimized and tailored to various systems of interest by controlling the size and surface modification, i.e. functionalization of the nanostructured films (*“surface chemistry”*), owing to the exceptional versatility of the deposition techniques. In particular, in this work, we demonstrated that the size selectivity capabilities, the large surface area, the high electrical conductivity, and bio-compatibility of these films, may greatly contribute the successful detection of several compounds, i.e., inorganic molecules, and biological compounds, as neurotransmitters. For instance, here the great advantage concerns the detection of dopamine in the presence of ascorbic acid, which represents an interference problem very difficult to avoid for clinical and bio-medical investigations. Using TiO_2_-modified Si electrodes a large number of electroactive probes were detected with high selectivity, sensitivity, reproducibility, and long term stability.

In addition, using TiO_2_ nanostructured coatings, the poisoning of the conventional GC bare electrodes, by the products of dopamine reduction, was reduced consistently. This important result could be achieved changing many parameters during the deposition of TiO_2_ films, i.e., thickness, and surface functionalities [13–15]. These TiO_2_-modified Si electrodes, could open the way for their exploitation as a coating on implantable microelectrodes for *in-vivo* monitoring of brain activity. This aspect could significantly facilitate the understanding of chemical communication between neurons and the diagnoses of malfunctions of the nervous system (i.e., Parkinsos’s disease) directly inside the brain.

## Figures and Tables

**Table 1.** Analytical parameters for TiO_2_-modified Si electrodes toward dopamine, epinephrine, and norepinephrine detection. Differential Pulse Voltammetric calibrations in 0.1 M phosphate buffer, pH 7.4 (20 mL) + 1 mM AA.

**Neurotransmitters**	**Linear range of concentration**	**Linear regression equations**	**LOD**	**RSD**	**Sensitivity**	**Response time**

	M	I_pa_/(μA) vs. C/(μM)	(S/N) = 3	(N = 30)	μA cm^−2^ μM^−1^	s

			M	(%)		
**Dopamine**	1.00 × 10^−8^ – 1.00 × 10^−4^	80.00 + 0.98 (r = 1.00)	1.00 × 10^−8^	1	122.50	10
**Epinephrine**	3.00 × 10^−8^ – 1.00 × 10^−4^	88.00 + 0.95 (r = 0.99)	3.00 × 10^−8^	2	118.75	12
**Norepinephrine**	5.00 × 10^−8^ – 1.00 × 10^−4^	90.00 + 0.97 (r = 0.98)	4.00 × 10^−8^	4	121.25	11

**Table 2.** Analytical parameters for GC bare electrodes toward dopamine, epinephrine, and norepinephrine. Differential Pulse Voltammetric calibrations in 0.1 M phosphate buffer, pH 7.4 (20 mL) + 0.01mM AA.

**Neurotransmitters**	**Linear range of concentration**	**Linear regression equations**	**LOD**	**RSD**	**Sensitivity**

**GC bare electrode**	M	I_pa_/(μA) vs. C/(μM)	(S/N) = 3	(N = 10)	μA cm^−2^ μM^−1^

			M	(%)	
**Dopamine**	3.7 × 10^−7^ – 1.0 × 10^−4^	1.522 + 0.154	3.0 × 10^−7^	3	18.0
**Epinephrine**	7.5 × 10^−7^ − 1.0 × 10^−4^	0.989 + 0.106	5.0 × 10^−7^	5	11.7
**Norepinephrine**	8.4 × 10^−7^ − 1.0 × 10^−4^	0.081 + 0.125	7.4 × 10^−7^	6	12.3
